# Environmental factors affecting childhood diarrheal disease among under-five children in Jamma district, South Wello zone, Northeast Ethiopia

**DOI:** 10.1186/s12879-019-4445-x

**Published:** 2019-09-13

**Authors:** Getachew Yismaw Workie, Temesgen Yihunie Akalu, Adhanom Gebreegziabher Baraki

**Affiliations:** 1 South Wello Zonal Health Department Public Health Emergency Early Warning and Preparedness Officer, South Wello, Ethiopia; 2 Department of Epidemiology and Biostatistics, Institute of Public Health, College of Medicine and Health Sciences, University of Gondar, Gondar, Ethiopia

**Keywords:** Childhood, Diarrhea, Jamma district

## Abstract

**Background:**

Globally, diarrhea is the leading cause of morbidity and mortality among less than 5 years old children and it contributes to the deaths of approximately one million children every year. In Ethiopia, diarrhea is the second cause of under-five mortality and morbidity. However, in the study area, studies were limited. Therefore, this study has assessed the prevalence of diarrhea and associated factors among < 5 years of age in Jamma district, Northeast Ethiopia.

**Methods:**

A community-based cross-sectional study was conducted from August 15 to September 15, 2017, in Jamma district, South Wello zone, northeast Ethiopia. A Systematic random sampling technique was used to select 614 households and a pretested structured questionnaire was used to collect the data. A multivariable logistic regression analysis was used to investigate factors associated with diarrheal disease. Adjusted Odds Ratio (AOR) with the corresponding 95% Confidence Interval (CI) for variables with *P*-value < 0.05 was used to show statistically significant association.

**Results:**

In this study, the prevalence of diarrhea among under-five children was 23.1% (95% CI: (19.4, 26.5). Child’s age 6 to 23 months [AOR: 2.46, 95% CI: (1.49, 4.05)], Living in rural area [AOR: 2.75, 95% CI: (1.33,5.66)], absence of latrine [AOR: 4.80, 95% CI: (2.39,9.60)], absence of handwashing facility [AOR: 2.45, 95% CI: (1.53,3.93], unprotected drinking water source [AOR:2.68, 95% CI: (1.54,4.68)], and Improper waste disposal practices [AOR:3.86, 95% CI: (2.38,6.26)] were associated with diarrhea disease.

**Conclusion:**

There was a high prevalence of diarrheal disease among children in the study area. Child age, rural residence, availability of latrine and handwashing facility, source of drinking water, and improper waste disposal were notably associated with childhood diarrheal disease. Therefore, improving handwashing practices and pure water supply, proper waste disposal including the availability of latrines would minimize the burden of diarrheal disease.

## Background

Globally, there are nearly 1.7 billion cases of childhood diarrhoeal disease every year [1]. Approximately 84% of the global burden of diarrheal disease is experienced by children under the age of 5 years [2]. Children of low and middle-income countries carry the highest proportion of this disease burden. In Africa diarrhea account for the largest cause of disease and death among young children and nearly 50% of deaths due to diarrhea among young children occurs in Africa [3]. A total of five episodes of diarrhea occur every year in a child living in Africa and 800,000 deaths occur due to diarrhea and dehydration [4]. One-fourth to the three-fourth proportion of childhood illness is due to diarrhea and 14% of children’s outpatient visits are due to this problem. Diarrhea exposes children to several other infections by predisposing them to malnutrition. It also accounts for 16% of hospital admissions malnutrition [5, 6].

Several factors affect the occurrence of diarrhea; these include child’s age, maternal education, household income, hygiene of feeding practices, breastfeeding status, malnutrition, personal hygiene, environmental sanitation, water availability and quality, and latrine utilization [7–9].

Evidence about the magnitude of diarrheal disease and the significant predictors in the study area was scarce, so this study was aimed to determine the prevalence and factors associated with childhood diarrheal disease.

## Methods

### Study design and period

A community-based cross-sectional study was conducted from August 15 to September 15, 2017.

### Study area and population

Jamma district is one of the 21 districts of South Wello administrative zone found in Amhara National Regional State, Ethiopia. Based on the 2007 population and housing census, Jamma has a total estimated population of 144,409. Of the total population, under-five children constitutes 19,784 (13.7%) and 131,399 (90.9%) of population lives in rural areas. The district has 6 health centers and 22 health posts. The Study populations were all households with at least one under-five child.

### Sample size and sampling procedure

A final sample size of 614 was determined using the assumption of *P* = 0.23 which is taken from a similar study [9], a margin of error 5%, the Z value of 1.96 for 95% Confidence Interval (CI), design effect of 2 and 10% contingency. Among 23 kebeles six kebeles were selected randomly and 20 gots (smaller administrative units) from a total of 60 gots in the 6 selected kebeles were randomly selected. All households that have at least one child were included in the study. In the case of the presence of more than one under-five children, lottery method was used to choose one child per household.

### Variable of the study

The dependent variable, diarrhea was defined as the presence of loose or watery stool ≥3 times during 24 h as reported by the mother/caregiver in the past 2 weeks before the survey. Independent variables like socio-demographic: family income, family size, number of children, parental education, parental occupation, marital status, sex of the child, age of child, maternal age, place of residence and religion; behavioral factors: water drawing and storage method, handwashing practice, feeding practice, and duration of breastfeeding; environmental factors: type of water source, distance of the water source, amount of daily water consumption, availability and functionality of latrine, presence of livestock in the house, and other factors like nutritional status of the children were used to assess diarrhea morbidity in the district. A water source is considered unprotected sources when there is no barrier or other structure to protect the water from contamination.

### Data collection procedures

A structured questionnaire was used to collect the data. Mothers/caregivers were interviewed on the occurrence of diarrheal disease within the past 2 weeks prior to the data collection. The nutritional status of the children was determined by mid-upper arm circumference (MUAC) for children aged between 12 and 59 months. Child length was measured on lying down (recumbent) position for children under the age of 2 years and height was used for children beyond 2 years.

### Data management and analysis

Data were cleaned, coded, and entered to Epi-info version 7 and transferred to SPSS for analysis. Summary measures like mean were calculated for continuous variables. Variables with *P*-value < 0.2 were entered for multivariable analysis. Variables with *P*-value < 0.05 with a 95% confidence interval were used to identify significant factors of diarrheal disease. The Adjusted odds ratio (AOR) was used to measure the strength of association and goodness of fit of the model was checked by Hosmer and Lemeshow test.

## Results

### Socio-demographic characteristics

A total of 614 households were included in the study with a response rate of 100%. More than 86% of households had only one under-five child in the family and the mean family size was 4.8 (±1.56SD) persons. The larger proportion of respondents 586 (95.4%) were biological mothers. Of the total 565 (92%), and 582 (94.9%) were married and housewives, respectively. Regarding religion, 341 (55%) were Christians. The mean age of the mothers/caregivers was 29.8 (±6.4) years **(**Table 1**)**.

**Table 1:** Socioeconomic characteristics of the respondents, Jamma district, Northeast Ethiopia, 2017

Variables	Frequency	Percentage (%)
Family size (persons per household)		
Less than five	306	49.8
Five and above	308	50.2
Number of under five children		
One	533	86.8
Two or more	81	13.2
Residence		
Rural	545	88.8
Urban	69	11.2
Relation of the respondent to the child		
Mother	586	95.4
Caregiver	28	4.6
Age of the mother/caretaker (in years)		
< 25	165	26.9
25–34	296	48.2
> 35	153	24.9
Marital status of mother		
Married	565	92
Single	10	1.6
Divorced	29	4.7
Widowed	8	1.3
Religion		
Muslim	273	44.5
Christian	341	55.5
Educational level of mother		
Unable to read and write	392	63.8
Primary	156	25.4
Secondary and higher	66	10.7
Occupation of the mother		
House wife	582	94.9
Governmental employee	22	3.5
Private	10	1.6
Educational level of father		
Unable to read and write	149	24.3
Primary	364	59.3
Secondary and above	101	16.4
Occupation of the father		
Farmer	519	84.5
Government employee	30	4.9
Merchant	65	10.6

### Environmental and behavioral characteristics

Five hundred sixty (91.2%), had floors made of mud/sand/dug, the majority of the households, 441 (71.8%) had a latrine. Regarding their source of water, 388 (63.2%) of households got from protected spring and pipe water. Most of the households, 337(54.9%), dispose of waste in an open dump. There was no handwashing facility in 355 (57.8%) of the households.

Out of 614 respondents, 584 (95.1%) respondents have used a container with narrow opening store water. Six hundred seven (98.9%) respondents used a covered container to fetch water. Most of the respondents, 472 (76.2%) took water from drinking storage container by pouring **(**Table 2**)**.

**Table 2:** Environmental and behavioral characteristics of respondents, Jamma district, Northeast Ethiopia, 2017

Variables	Frequency	Percentage (%)
Types of roof material of the living house		
Thatched	41	6.7
Corrugated iron sheet	573	93.3
Types of floor material of the living house		
Mud/sang/dug	560	91.2
Cement	46	7.5
Wood	8	1.3
Animals live with family in one house		
Yes	96	15.6
No	518	84.4
Number of rooms in the house		
One	77	12.5
Two	223	36.3
More than two	314	51.1
Availability of latrine		
Yes	441	71.8
No	173	28.2
Availability of handwashing facility		
Yes	259	42.2
No	355	57.8
Main source of water		
Protected	388	63.2
Unprotected	226	36.8
Distance of water source		
< 30 min	193	31.4
> 30 min	421	69.6
Way of taking water from container		
Pouring	472	76.2
Dipping	142	23.1
Site of waste disposal		
Pit/Burn	277	45.1
Open dump	337	54.9
Breastfeeding status		
No	176	28.6
Partial	346	56.4
Exclusive	92	15
Child feeding methods/material		
Hand	295	48
Cup and spoon	227	36.9

### Child demographics, nutritional and health characteristics

There were slightly more male 330 (53.7%) children than females. The mean age of the children was 21.9 (SD ± 14.3) months. The majority of children, 346 (56.4%), were partially breastfed **(**Table 3**)**.

**Table 3:** Demographic, nutritional and health characteristics of the index children in Jamma district, Northeast Ethiopia, 2017

Variables	Frequency	Percentage (%)
Age of the child		
1–5 months	52	8.5
6–23 months	338	55
24–59 months	224	36.5
Sex of the child		
Male	330	53.7
Female	284	46.3
Place of birth		
Health Institution	517	84.2
Home	97	15.8
Birth Order		
First	156	25.4
Second – third	320	52.1
Fourth and above	138	22.5
Nutritional status of the child		
Malnourished	53	8.6
Well nourished	561	91.4
Number of Rota vaccine received		
1 drop	409	66.6
2 drop	188	30.6
3 drop	17	2.8

### Prevalence of diarrheal disease

Findings from this study showed that 142 children had experienced diarrhea in the last 2 weeks preceding the survey, giving a prevalence of 23.1% (95% CI, 19.4–26.5%).

### Factors affecting childhood diarrhea

In multivariable logistic regression child’s age, residence, availability of latrine, availability of handwashing facility, source of water, and waste disposal practice were independently associated with diarrheal disease.

Children aged 6 to 23 months had 2.46 [AOR: 2.46, 95%CI (1.49, 4.05)] times higher odds of diarrhea compared to children less than 6 months The odds of developing diarrhea among rural children were 2.75 [AOR: 2.75, 95%CI: (1.33, 5.66)] times compared to their counterparts. Children from households with no latrine facility had 4.8 [AOR: 4.8, 95% CI (2.39, 9.60)] times higher odds of developing diarrhea than children from households who had latrine facilities. The odds of developing diarrhea was 2.45 [AOR: 2.45, 95% CI: (1.53, 3.93)] times higher among children whose households had no handwashing facility compared to their counterparts. Children with unprotected drinking water source had 2.68 [AOR: 2.68, 95% CI: (1.54, 4.68)] times higher odds of diarrhea than children with protected water sources. Children with openly dumped waste around the house had 3.86 [AOR: 3.86, 95% CI (2.38, 6.26)] times higher odds of diarrhea compared to their counterparts **(**Table 4**)**.

**Table 4:** Factors affecting diarrhea disease among children under 5 years of age, Jamma district, Northeast Ethiopia, 2017

Variables	Diarrhea		COR (95%) CI	AOR(95%)CI
	Yes	No		
Residence				
Rural	116	429	2.24 (1.32–3.8)	2.75 (1.33–5.66)*
Urban	26	43	1	1
Educational level of mother				
Unable to read and write	49	343	2.6 (1.4–4.9)	1.72 (0.77–3.83)
Primary	75	81	0.4 (0.21–0.76)	0.29 (0.13–0.66)
Secondary and higher	18	48	1	1
Types of floor material of the living house				
Mud/sand/dug	120	440	3.1 (1.66–5.69)	2.46 (0.96–6.31)
Wood	1	7	5.88 (0.67–51.7)	5.45 (0.19–154)
Cement	21	25	1	1
Availability of latrine /toilet facilities				
Yes	130	311	1	1
No	12	161	5.6 (3.01–10.44)	4.80 (2.39–9.60)*
Availability of handwashing facilities				
Yes	94	165	1	
No	48	307	3.6 (2.45–5.40)	2.45 (1.53–3.93)*
Main source of drinking water				
Protected	117	271		
Unprotected	25	201	3.47 (2.17–5.55)	2.68 (1.54–4.68)*
Site of waste disposal				
Pit/burn	99	178	1	
Open dump	43	294	3.8 (2.54–5.69)	3.86 (2.38–6.26)*
Way of taking water from container				
Pouring	126	346	1	1
Dipping	16	126	2.9 (1.64–5.0)	4.0 (0.86–7.90)
Age of index child				
1–5 months	11	41	1.8 (0.88–3.7)	1.71 (0.98–2.66)
6–23 months	58	280	2.33 (1.57–3.47)	2.46 (1.49–4.05)*
> 24 months	73	151	1	1

*P-value < 0.05

## Discussion

This study determined the magnitude of diarrhea and the factors affecting it. The 2 week prevalence of diarrhea was 23.1% and a child’s age, residence, availability of latrine, availability of handwashing facility, source of water, and waste disposal practice were independently associated with diarrheal disease.

The prevalence of diarrhea in this study (23.1%) was higher than the Ethiopian national prevalence of diarrheal disease (13%) as reported by EDHS 2016 [10]. It is also higher than a study conducted in KeffaSheka [11], Amhara region [7], rural Tanzania [12] and Bangladesh [13]. This figure was in line with a study from northwest Ethiopia [8] and Cameroon (23.8%) [9] However, it was lower when compared with some parts of the country (Ethiopia), which was 33.7% at Nekemte town [14], and 30.5% at Arbaminch [15]. The possible reason could be variation in the distribution of water supply, health, and other facilities across these different settings.

The odds of having diarrhea was higher among rural children than urban ones and this finding was in line with the findings in some parts of Ethiopia like Kersa, Debrebirhan town and Jabithennan [8, 16, 17]. This could be related to the wide discrepancy in the presence of infrastructures that affect the occurrence of diarrhea, these include health care, water and sanitation facilities and literacy [18].

The finding of this study showed that children aged 6 to 23 months were at high risk of developing diarrhea than children 2 years old. This finding is in agreement with other studies conducted in Arbaminch and Benishangul Gumuz, and districts of the Amhara region [7, 15, 19]. Children above the age of 6 months are at the age where they are introduced to foods other than breast milk, this may expose their undeveloped immunity to infectious agents causing diarrhea. Besides children at these ages will start to crawl, thus they may pick dirt or other contaminated objects and take to their mouth.

Open waste disposal around the house was also found to be a significant risk factor for diarrhea. This finding was in line with studies conducted in Sheko district and Kersa eastern Ethiopia [16, 20]. Open waste disposal causes the child to contact to contaminated environment and also creates an ideal environment for flies that carry the pathogens to water, food and food utensils.

This study found a significant association between diarrheal disease and lack of latrine which is supported by another study conducted in Derashe town [21], northwest Ethiopia [7] and Ghana [22]. The simple explanation might be that the availability of latrine reduces fecal contamination of the environment and also it reduces the chance of mechanical vectors’ access to diarrhea-causing organisms thereby reducing diarrheal disease.

The finding of our study showed that the use of unprotected water sources was significantly associated with diarrheal disease. This study is consistent with the study Derashe district, Southern Ethiopia [21] and Pawi Special District in Benishangul-Gumuz Region [23]. Since unprotected sources are those with no barrier or other structure to protect the water from contamination; they can get contaminated easily and cause diarrhea while ingested. Unprotected water sources are also important source of diarrhea causing intestinal parasites like giardiasis [23].

This study can be generalized to all under-five children in Jamma district and for other areas with similar setting however; it shares the limitation of a cross-sectional study. As a result, this study may have a difficulty to show the temporal relationship between exposure and outcome variable.

## Conclusion

In conclusion, the findings of this study showed that the prevalence of childhood diarrheal disease was high. So, childhood diarrheal disease remains a serious public health challenge in the study area. Living in rural areas, lack of sanitation facilities, unprotected sources of drinking water, improper waste disposal, and child age were significantly associated with childhood diarrheal disease. Therefore, improving handwashing practices and pure water supply, proper waste disposal including the building and utilizing latrines would minimize the burden of diarrheal disease.

## Data Availability

Data will be available from the corresponding author upon a reasonable request.

## Abbreviations

CSA: Central Statistical Agency
EDHS: Ethiopia Demographic Health Survey
ETB: Ethiopian Birr
GC: Gregorian calendar
SPSS: Statistical Package for Social Studies
WHO: World Health Organization
